# Synergic Elevation of Systemic Inflammation by the Coexistence of Periodontitis and Diabetes Mellitus: A Nationwide Analysis of Korean Adults

**DOI:** 10.3390/biomedicines13102441

**Published:** 2025-10-07

**Authors:** Hye-Sun Shin

**Affiliations:** Department of Dental Hygiene, Dongnam Health University, Suwon-si 16328, Gyeonggi-do, Republic of Korea; prevoralhealth@gmail.com

**Keywords:** diabetes mellitus, high-sensitivity C-reactive protein, Korean National Health and Nutrition Examination Survey, periodontitis, systemic inflammation

## Abstract

**Background/Objectives:** This study aimed to evaluate the additive effect of periodontitis and diabetes mellitus on systemic inflammation, measured by high-sensitivity C-reactive protein (hs-CRP), in a nationally representative Korean population. **Methods:** Data from 3178 adults (≥19 years) in the 2015 Korean National Health and Nutrition Examination Survey were analyzed. Periodontitis was assessed using the Community Periodontal Index (CPI), and diabetes mellitus was defined based on clinical criteria. Participants were classified into four groups according to the presence of periodontitis and diabetes. hs-CRP levels were analyzed by quartiles and ADA/CDC cardiovascular risk categories. ANCOVA and multivariable logistic regression, adjusted for socioeconomic status, oral health and health behaviors, and comorbidities, were used to examine the association between coexisting periodontitis and diabetes and elevated hs-CRP. **Results:** Mean hs-CRP increased progressively from G1 (1.11 ± 0.49 mg/L) to G4 (2.37 ± 0.38 mg/L). After adjustment, G4 retained the highest concentration (2.31 ± 0.39 mg/L) versus G1 (1.37 ± 0.11 mg/L; *p* = 0.020). High-risk hs-CRP prevalence (>3.0 mg/L) increased nearly threefold across groups (*p* < 0.001). Similarly, G4 had increased odds of being in the ADA/CDC high-risk category (>3.0 mg/L) (aOR = 2.73, 95% CI: 1.64–4.54), whereas no significant associations were observed for periodontitis or diabetes alone. **Conclusions:** The coexistence of periodontitis and diabetes mellitus is significantly associated with elevated systemic inflammation, as measured by hs-CRP, suggesting a synergistic effect beyond the impact of either condition alone.

## 1. Introduction

Periodontitis and type 2 diabetes mellitus are two of the most prevalent chronic inflammatory diseases worldwide, with severe periodontitis affecting over 1 billion individuals in 2021 and projected to rise substantially by 2050 [1,2]. Recent analysis by the Non-Communicable Disease Risk Factor Collaboration found that more than 800 million adults worldwide have diabetes, with global prevalence doubling from 7% to 14% between 1990 and 2022 [3,4].

Both conditions share a common mechanistic pathway of chronic systemic inflammation, characterized by elevated levels of pro-inflammatory mediators such as interleukin-1β, interleukin-6, tumor necrosis factor-α, and C-reactive protein CRP [5]. High-sensitivity CRP (hs-CRP) has emerged as a sensitive biomarker of systemic inflammation and cardiovascular risk [5,6]. A recent meta-analysis on advanced biomarkers for predicting incident cardiovascular disease reported that higher baseline hs-CRP levels are significantly associated with future cardiovascular events, with a pooled hazard ratio of 1.19 per unit increase, reinforcing hs-CRP’s value as a sensitive predictor of cardiovascular risk in the general population [6].

A recent systematic review and meta-analysis, based on 15 cohort studies, demonstrated a positive bidirectional association between periodontitis and diabetes mellitus, with a moderate certainty of evidence [7]. Among patients with diabetes, the incidence of periodontitis was increased by 24%. Conversely, individuals with periodontitis had a 26% higher risk of developing diabetes. These findings are consistent with those of the meta-analysis conducted by Nascimento et al. [8] which reported an 86% increased relative risk of periodontitis in individuals with diabetes. Also, recent findings based on data from the Korea National Health and Nutrition Examination Survey (KNHANES) revealed that incident diabetes mellitus showed stronger associations with Community Periodontal Index (CPI) scores of 3 and 4 than did pre-existing DM, suggesting that advanced periodontal disease may increase the risk of developing DM [9,10].

Although randomized controlled trials [11,12,13]—including those conducted in recent years—have consistently demonstrated that periodontal therapy can improve glycemic control, typically reflected by reductions in HbA1c and, less frequently, in hs-CRP, there remains a significant gap in epidemiological understanding of how the coexistence of periodontitis and diabetes influences systemic inflammation. Most intervention studies have focused on isolated treatment effects, without systematically comparing the inflammatory burden across different combinations of periodontal and diabetic status.

Despite the findings of previous randomized controlled trials, there remains a lack of epidemiological studies that systematically compare how different combinations of periodontitis and diabetes status influence hs-CRP levels. It is necessary to examine whether and how systemic inflammation varies according to the coexistence patterns of these two conditions.

Therefore, this study aimed to investigate the association between hs-CRP levels and the presence of periodontitis, diabetes mellitus, and their co-existence. Participants were stratified into groups based on disease status, and hs-CRP levels were categorized using multiple established thresholds to enable a quantitative assessment of systemic inflammation.

hs-CRP was used as the primary outcome to evaluate the additive effect of periodontitis and diabetes mellitus on systemic inflammation in a nationally representative Korean dataset. By applying complex survey design procedures and adjusting for key confounders—including age, sex, socioeconomic status, health behaviors, and comorbid conditions—this study seeks to address a critical methodological and conceptual gap and to inform integrated clinical management strategies aimed at reducing inflammatory and cardiovascular risk. Additionally, we sought to evaluate whether the coexistence of periodontitis and diabetes mellitus is associated with significantly increased odds of elevated hs-CRP levels—particularly within the highest cardiovascular risk category—as compared to having either condition alone, thereby addressing a critical gap in stratified population-level analyses.

## 2. Materials and Methods

### 2.1. Data Sources and Sampling Design

This study analyzed the 2015 KNHANES, a continuous, nationally representative cross-sectional survey overseen by the Korea Disease Control and Prevention Agency under the National Health Promotion Act. KNHANES employs a clustered, stratified, multistage probability sampling design to capture approximately 10,000 non-institutionalized Korean residents aged ≥1 year each year. In each survey cycle, 192 Primary Sampling Units (PSUs) are selected from about 200,000 geographically defined areas across Korea. Within each PSU, 20 households are chosen via systematic sampling (yielding ~3840 households), and all members aged ≥1 year in selected households are eligible to participate. Sample weights account for the complex survey design, nonresponse, and post-stratification by age and sex to ensure national representativeness.

### 2.2. Survey Components & Data Collection

The KNHANES comprises three integrated components: the Health Interview, which gathers information through face-to-face interviews and self-administered questionnaires on demographics, socioeconomic status, health behaviors such as smoking, alcohol use, and physical activity, as well as mental health, oral health, disease morbidity, and healthcare utilization; the Health Examination, conducted by trained medical personnel at mobile centers, includes anthropometric measurements, blood pressure, laboratory tests (blood and urine), periodontal assessment, and other clinical evaluations following standardized protocols with routine quality-control calibration; and the Nutrition Survey, in which dietitians visit households approximately one week later to assess dietary behaviors, conduct 24-h dietary recalls, evaluate food frequency, and examine food security.

### 2.3. Ethical Approval

Written informed consent was obtained from all participants. Since 2015, the KNHANES has been conducted without deliberation from the institutional review board, which corresponds to research conducted by the government to ensure guidelines meet public welfare principles in accordance with the Bioethics Act.

### 2.4. Study Participants

A total of 7380 individuals participated in the 2015 KNHANES. Participants were excluded if they lacked data on the primary independent variables (periodontitis and diabetes diagnoses) or high-sensitivity CRP measurements, as well as if they had missing values for any covariates. After applying these exclusion criteria, the final analytic sample comprised 3189 (1351 men and 1827 women) participants.

### 2.5. Assessment of Periodontitis

The CPI was recorded using a 0.5 mm ball-tipped probe and the walking probing technique at a standardized force of ~20 g. Dentition was divided into six sextants (18–14, 13–23, 24–28, 38–34, 33–43, 44–48) and assessed at ten index teeth (#17, 16, 11, 26, 27, 37, 36, 31, 46, 47), substituting adjacent teeth if missing. Each sextant was scored 0–4: 0 = healthy; 1 = bleeding; 2 = calculus; 3 = PPD 3.5–5.5 mm; 4 = PPD > 5.5 mm. A participant was classified as having periodontitis if any sextant scored ≥ 3; otherwise, they were deemed periodontally healthy.

### 2.6. Assessment of hs-CRP Levels

Serum hs-CRP concentrations were measured by certified reference laboratories using standardized calibration procedures as part of the KNHNES. For statistical analysis, hs-CRP values were utilized in three distinct ways: (1) as a continuous variable; (2) categorized into quartiles based on the distribution of hs-CRP levels in the study population (quartile I: 0–0.30 mg/L, quartile II: 0.31–0.55 mg/L, quartile III: 0.56–1.00 mg/L, quartile IV: 1.10–21 mg/L); and (3) classified into three clinical risk categories—low risk (<1.0 mg/L), average risk (1.0–3.0 mg/L), and high risk (>3 mg/L)—according to the criteria established by the American Diabetes Association and the Centers for Disease Control and Prevention (ADA/CDC) [5].

### 2.7. Assessment of Diabetes Mellitus

The diabetes variable (among participants fasting ≥8 h) was categorized into three groups:

- Normal glucose regulation: fasting plasma glucose < 100 mg/dL and HbA1c < 5.7%.
- Prediabetes: fasting plasma glucose 100–125 mg/dL or HbA1c 5.7–6.4%.
- Diabetes: fasting plasma glucose ≥ 126 mg/dL, or prior physician diagnosis of diabetes, or current use of glucose-lowering medications (oral hypoglycemic agents or insulin), or HbA1c ≥ 6.5%.

For the analysis, the diabetes variable was categorized into two groups: individuals without diabetes and individuals with diabetes.

### 2.8. Assessment of Potential Confounders

The diabetes variable (among participants fasting ≥8 h) was categorized into three groups: For the analysis, the following confounders were included as adjustment variables: age, gender, household income, education, oral health-related behaviors (smoking status, alcohol consumption, and aerobic physical activity), and systemic conditions (obesity, hypertension, and hypercholesterolemia). Education was categorized into four groups: primary school, middle school, high school, and college or university. Household income was classified into quartiles according to equivalized monthly income to reflect socioeconomic status. Smoking status was dichotomized as never/former smokers versus current smokers, and alcohol consumption was classified as either no alcohol intake in the past month or any alcohol consumption at least once during the past month. Aerobic physical activity was defined according to international guidelines as engaging in at least 150 min per week of moderate-intensity exercise—such as brisk walking or dancing—or 75 min per week of vigorous-intensity exercise—such as running or fast cycling—or an equivalent combination of both, in which each minute of vigorous-intensity activity is counted as two minutes of moderate-intensity activity, yielding a total of at least 150 “moderate-equivalent” minutes weekly. Hypertension was characterized by a systolic blood pressure of 140 mmHg or higher, a diastolic blood pressure of 90 mmHg or higher, or the current use of antihypertensive medication. Obesity was determined using the body mass index (BMI), calculated as weight in kilograms divided by the square of height in meters, with a BMI threshold of 25.0 kg/m^2^ or greater indicating obesity. Hypercholesterolemia was defined as having a total serum cholesterol level exceeding 240 mg/dL or the use of lipid-lowering medications.

### 2.9. Statistical Analysis

All statistical analyses were conducted using SPSS version 29.0 (IBM Corp., Armonk, NY, USA), employing complex sampling techniques based on the survey design and in accordance with the official analytical guidelines for KNHANES data. All analyses incorporated the complex survey design, including sampling weights, stratification, and clustering, to ensure nationally representative estimates [14]. The general characteristics of variables according to the presence or absence of periodontitis and diabetes mellitus were analyzed as follows. Demographic, socioeconomic, behavioral, and systemic variables were compared across groups defined by periodontitis and diabetes status. Categorical variables were presented as weighted percentages with standard errors, and group differences were assessed using the chi-square test. Continuous variables were summarized as means with standard errors and compared between groups using the *t*-test. Statistical significance was determined at a *p*-value of less than 0.05. Categorical variables, such as hs-CRP quartile and ADA/CDC risk categories, were summarized as weighted counts and percentages. Group differences were evaluated using the chi-square test to determine statistical significance. For continuous variables, such as mean hs-CRP concentration, values were reported as means with standard errors, and differences between groups were assessed using the *t*-test. After then, participants were classified into four groups based on the presence or absence of periodontitis and diabetes: G1 (neither condition), G2 (periodontitis only), G3 (diabetes only), and G4 (both conditions). Hs-CRP concentrations were analyzed in two ways: by population quartiles and by clinical risk categories. Categorical variables, such as hs-CRP quartiles and risk categories, were summarized as weighted percentages with standard errors, and differences between groups were evaluated using the chi-square test. For continuous variables, such as mean hs-CRP levels (both unadjusted and adjusted), values were reported as means with standard errors, and group differences were assessed using analysis of variance (ANOVA) for unadjusted comparisons and analysis of covariance (ANCOVA) for adjusted comparisons. The adjusted analyses controlled for potential confounders, including age, gender, income, education, smoking, alcohol consumption, physical activity, hypertension, obesity, and hypercholesterolemia. Finally, multivariable logistic regression analyses were conducted to estimate the adjusted odds ratios (ORs) and 95% confidence intervals (CIs) for elevated hs-CRP levels across the four periodontitis–diabetes coexistence groups (G1–G4). Analyses were performed separately for hs-CRP quartiles and ADA/CDC cardiovascular risk categories, using the group without periodontitis and diabetes (G1) as the reference. All models were adjusted for age, sex, household income, education level, smoking status, alcohol consumption, physical activity, toothbrushing frequency, regular dental visit, obesity, hypertension, and hypercholesterolemia.

## 3. Results

### 3.1. Baseline Characteristics of Study Participants

Table 1 presents descriptive statistics according to the presence or absence of periodontitis and diabetes mellitus. Among all study participants, an estimated 34.3% had periodontal disease and 11.2% had diabetes mellitus. Participants with periodontitis or diabetes tended to be older and had lower income and educational attainment compared to those without these conditions. Toothbrushing frequency of less than twice per day was significantly more common in participants with periodontitis (12.4%) and diabetes mellitus (14.7%) compared to those without these conditions (8.1% and 8.9%, respectively; *p* < 0.01 for both), while regular dental visit frequency did not significantly differ between groups. The prevalence of hypertension, obesity, and hypercholesterolemia was significantly higher among groups with periodontitis and/or diabetes. Current smoking and alcohol consumption rates were notably elevated in the periodontitis group, while physical inactivity was more frequent in the diabetes group. Statistically significant differences were observed for most demographic, behavioral, and clinical characteristics across groups, highlighting that individuals with periodontitis and diabetes are more likely to have adverse health profiles and multiple risk factors.

### 3.2. Systemic Inflammatory Markers According to Periodontitis and Diabetes

Table 2 shows that higher hs-CRP levels were significantly associated with both periodontitis and diabetes mellitus. Participants with periodontitis or diabetes had a greater proportion in the highest hs-CRP quartile and high-risk ADA/CDC category compared to those without these conditions. Mean hs-CRP concentrations were also significantly higher in groups with periodontitis or diabetes. These differences remained statistically significant (*p* < 0.001), indicating that both periodontitis and diabetes are linked to elevated systemic inflammation.

### 3.3. Hs-CRP Levels According to the Co-Existence of Periodontitis–Diabetes

Table 3 presents the most significant results, highlighting the additive effect of periodontitis and diabetes mellitus on systemic inflammation as measured by hs-CRP concentrations. After classifying all participants into four groups, the distribution was as follows: 60.1% in Group 1 (neither periodontitis nor diabetes), 5.6% in Group 2 (periodontitis only), 28.6% in Group 3 (diabetes only), and 5.6% in Group 4 (both periodontitis and diabetes). When examining the distribution of hs-CRP quartiles, it was evident that as the quartile increased, the proportion of participants in Groups 3 (diabetes only) and 4 (both periodontitis and diabetes) became markedly higher. In the highest hs-CRP quartile (Q4), these two groups accounted for a substantially greater share compared to Groups 1 and 2 (*p* < 0.001). This pattern indicates that individuals with diabetes—especially those who also have periodontitis—are more likely to exhibit elevated systemic inflammation, as reflected by higher hs-CRP levels. The distribution of hs-CRP levels classified into three categories according to ADA/CDC criteria—low risk (24.5%), average risk (42.6%), and high risk (32.9%)—closely mirrored the patterns observed with the quartile-based analysis, and these differences were also statistically significant (*p* < 0.001). When hs-CRP was analyzed as a continuous variable, significant differences in mean concentrations were observed across all groups (*p* = 0.001). After adjusting for all potential confounders, Groups 3 and 4 exhibited significantly higher hs-CRP levels compared to Groups 1 and 2, indicating a statistically significant elevation in systemic inflammation among participants with diabetes, particularly those with both diabetes and periodontitis (*p* = 0.021).

### 3.4. Association Between Periodontitis–Diabetes Co-Existence and Elevated hs-CRP Risk

After adjusting for potential confounders, the odds of being in the highest hs-CRP quartile (Q4, 1.10–21 mg/L) were significantly elevated in individuals with both periodontitis and diabetes mellitus (G4), with an adjusted odds ratio (aOR) of 2.54 (95% CI: 1.39–4.68) compared to the reference group (G1: no periodontitis, no diabetes). No statistically significant associations were observed for the other groups (G2 and G3) across the quartiles (Table 4). When hs-CRP was categorized according to ADA/CDC cardiovascular risk levels, individuals in the G4 group had 1.77 times higher odds of being in the average-risk category (1.0–3.0 mg/L) (95% CI: 1.14–2.75) and 2.73 times higher odds of being in the high-risk category (>3.0 mg/L) (95% CI: 1.64–4.54), both statistically significant compared to the reference group (G1). In contrast, no significant associations were found for G2 or G3 in either the average-risk or high-risk hs-CRP categories.

## 4. Discussion

This comprehensive analysis of 3178 Korean adults from the Korea National Health and Nutrition Examination Survey provides compelling evidence for the synergistic relationship between periodontitis and diabetes mellitus in elevating systemic inflammatory burden.

The stepwise increase in the representation of G3 and G4 across the quartiles underscores the additive effect of these conditions on inflammatory burden. The most striking finding was that individuals with both conditions exhibited the highest mean hs-CRP level (2.31 ± 0.39 mg/L), representing a statistically significant elevation compared to those with neither condition (1.37 ± 0.11 mg/L). This 72% increase in hs-CRP levels among patients with coexistent periodontitis and diabetes mellitus suggests a clinically meaningful amplification of cardiovascular risk that extends beyond the additive effects of individual conditions. Our analysis revealed that participants with both periodontitis and diabetes mellitus had 2.73 times higher odds of being classified in the high-risk hs-CRP category (>3.0 mg/L) (adjusted OR 2.73; 95% CI: 1.64–4.54), suggesting that the coexistence of these conditions substantially increases systemic inflammation and may critically elevate cardiovascular risk.

The observed synergistic elevation of hs-CRP in patients with concurrent periodontitis and diabetes mellitus can be attributed to several interconnected biological mechanisms. Periodontitis, characterized by chronic bacterial infection and subsequent host inflammatory response, triggers the release of pro-inflammatory cytokines including interleukin-1β (IL-1β), interleukin-6, and tumor necrosis factor-α (TNF-α) [15]. These cytokines not only perpetuate local periodontal destruction but also enter systemic circulation, contributing to hepatic C-reactive protein synthesis [16].

Simultaneously, diabetes mellitus creates a systemic pro-inflammatory milieu through multiple pathways. Hyperglycemia-induced oxidative stress, advanced glycation end products, and adipose tissue dysfunction all contribute to elevated circulating inflammatory mediators [17]. The metabolic dysfunction characteristic of diabetes mellitus also impairs immune cell function, potentially compromising the host’s ability to resolve periodontal inflammation effectively [18,19].

The convergence of these pathways creates a self-perpetuating cycle where periodontal inflammation exacerbates insulin resistance, while hyperglycemia impairs periodontal healing and immune function [20,21]. This bidirectional relationship explains why the inflammatory burden, as measured by hs-CRP, is not merely additive but rather multiplicative in patients with both conditions [7].

Our findings are consistent with a growing body of evidence demonstrating the interconnected nature of periodontal disease, diabetes, and cardiovascular risk. A comprehensive meta-analysis by Romero-Cabrera et al. [6] established hs-CRP as a significant predictor of cardiovascular events, with each unit increase associated with a 19% increase in risk (HR 1.19; 95% CI 1.09–1.30). Our population-level data supports this relationship by demonstrating that conditions known to elevate hs-CRP—namely periodontitis and diabetes mellitus—show synergistic effects when present concurrently.

Clinical trials by Oliveira et al. [11] have demonstrated that periodontal therapy can reduce both HbA1c and hs-CRP levels in patients with diabetes mellitus, providing mechanistic support for the bidirectional relationship observed in our cross-sectional analysis. However, most previous studies have focused on isolated treatment effects rather than quantifying the baseline inflammatory burden associated with disease coexistence [6,11,12,13].

Compared to previous studies that primarily focused on the risk of developing diabetes mellitus in relation to periodontitis, particularly among younger individuals [9,22], our study highlights a different perspective. We demonstrated that the co-existence of periodontitis and diabetes mellitus is significantly associated with elevated systemic inflammation, as measured by hs-CRP, and that this combined effect is more substantial than the presence of either condition alone. Unlike prior research emphasizing the predictive role of periodontitis for incident diabetes mellitus, our findings underscore the synergistic inflammatory burden when both conditions are present, suggesting the need for integrated management strategies targeting both periodontitis and diabetes mellitus. Importantly, no prior studies have systematically compared the full spectrum of periodontitis and diabetes mellitus co-existence across four distinct groups—those with neither condition, periodontitis only, diabetes mellitus only, and both periodontitis and diabetes mellitus. This absence of stratified analysis represents a critical gap in the literature. By addressing this gap, our study provides novel and clinically relevant evidence that the co-occurrence of periodontitis and diabetes mellitus is associated with a disproportionately elevated systemic inflammatory response, as measured by hs-CRP, beyond the additive effects of each condition alone.

High-sensitivity C-reactive protein has emerged as one of the most robust inflammatory biomarkers for cardiovascular risk prediction [5]. Unlike traditional risk factors that primarily reflect atherosclerotic burden, hs-CRP provides insight into the inflammatory component of cardiovascular disease pathogenesis [6]. The elevation observed in our study population with concurrent periodontitis and diabetes mellitus is particularly concerning given the established relationship between hs-CRP levels and future diabetes.

The synergistic relationship between periodontitis and diabetes mellitus in elevating hs-CRP levels provides compelling evidence for integrated treatment approaches that address both conditions simultaneously. An integrated approach would involve routine periodontal assessment as part of diabetic care protocols, with particular attention to inflammatory markers such as hs-CRP. Periodontal therapy, including scaling and root planing, antimicrobial treatment, and ongoing maintenance, should be considered an adjunctive component of diabetes management rather than a separate treatment modality.

The findings from this study have significant implications for public health policy and healthcare system organization. With the global prevalence of both severe periodontitis and diabetes projected to affect over 1 billion and 800 million individuals, respectively, by 2050 [1,2], the potential for synergistic inflammatory burden represents a substantial public health challenge. Public health education campaigns should emphasize the interconnected nature of oral health, diabetes management, and cardiovascular risk. This messaging could help bridge the traditional gap between dental and medical care, encouraging patients to view oral health as an integral component of overall health maintenance rather than a separate concern.

This study’s principal strength lies in its utilization of a large, nationally representative dataset with rigorous complex survey weighting that ensures generalizability to the Korean adult population. The comprehensive adjustment for potential confounders, including age, sex, education level, income, smoking status, and body mass index, enhances the validity of the observed associations. However, several limitations must be acknowledged. The cross-sectional design precludes the establishment of causal relationships and temporal sequencing between periodontal disease, diabetes, and inflammatory elevation. Longitudinal studies would be necessary to determine whether periodontal inflammation precedes diabetes onset or vice versa, and whether successful treatment of either condition can prevent or reverse the inflammatory burden. To fully elucidate these causal pathways, future research employing longitudinal or interventional designs is essential. A further limitation is the reliance on the Community Periodontal Index (CPI) for case ascertainment, which, as a partial-recording screening tool, may underestimate true disease severity compared to full-mouth clinical attachment level or probing depth measurements and introduce misclassification bias. Consequently, future research employing the 2018 World Workshop classification or the 2017 AAP/EFP case definitions may achieve more accurate periodontal assessments and stronger validity of observed associations [23,24]. In addition, while subgroup analyses could provide valuable insights, the sample size within relevant strata, particularly in the G4 group representing the coexistence of periodontitis and diabetes mellitus, was too small. Insufficient sample sizes in subgroups can lead to reduced statistical power, unstable effect estimates, and wide confidence intervals, which may undermine the reliability of the findings. To preserve the statistical validity of the results, subgroup analyses were not conducted in this study. Future research plans include merging additional national data sources to secure adequate sample sizes, enabling robust stratified analyses of key effect modifiers.

Notwithstanding these limitations, this study is distinguished by the use of hsCRP as a primary outcome variable, employing not only quartile-based analyses but also applying the ADA/CDC clinical cardiovascular risk thresholds. Furthermore, the study quantitatively assessed the synergistic effect of concomitant periodontitis and diabetes on systemic inflammation, demonstrating that the combined presence of both conditions is associated with a greater elevation in inflammatory burden than either condition alone. These methodological strengths enhance the clinical and epidemiological significance of the findings.

## Figures and Tables

**Table 1:** General characteristics according to periodontitis and diabetes mellitus (N = 3178).

	Periodontitis			Diabetes Mellitus		
Variables	No (*n* = 2089)	Yes (*n* = 1089)	*p*-Value *	No (*n* = 2821)	Yes (*n* = 357)	*p*-Value *
Age (years), mean ± SE	42.75 ± 0.50	54.59 ± 0.66	**<0.001 †**	45.17 ± 0.43	58.93 ± 0.83	**<0.001 †**
Gender						
Male	802 (45.3)	549 (55.0)	**<0.001**	1162 (47.5)	189 (56.0)	**0.015**
Female	1287 (54.7)	540 (45.0)		1659 (52.5)	168 (44.0)	
Income						
Lowest quartile	435 (21.2)	300 (27.8)	**0.005**	632 (22.4)	103 (31.5)	**0.007**
Lower middle quartile	497 (24.6)	278 (25.4)		685 (24.9)	90 (24.2)	
Upper middle quartile	578 (27.3)	262 (23.1)		766 (26.7)	74 (19.2)	
Highest quartile	579 (26.8)	249 (23.7)		738 (26.0)	90 (25.1)	
Education						
Primary school	377 (11.9)	321 (22.4)	**<0.001**	558 (13.3)	140 (34.6)	**<0.001**
Middle school	186 (6.5)	166 (14.0)		295 (8.3)	57 (14.1)	
High school	730 (37.5)	344 (34.9)		979 (37.4)	95 (29.4)	
College/University	796 (44.1)	258 (28.7)		989 (41.1)	65 (21.9)	
Smoking						
Non-smokers	1821 (83.8)	877 (76.6)	**<0.001**	2400 (81.8)	298 (79.7)	0.493
Current smokers	268 (16.2)	212 (23.4)		421 (18.2)	59 (20.3)	
Drinking						
No	538 (22.1)	354 (27.4)	**0.003**	757 (22.6)	135 (35.6)	**<0.001**
Yes	1551 (77.9)	735 (72.6)		2064 (77.4)	222 (64.4)	
Physical activity						
No	1043 (45.9)	614 (52.8)	**0.008**	1444 (47.0)	213 (58.0)	**0.001**
Yes	1046 (54.1)	475 (47.2)		1377 (53.0)	144 (42.0)	
Toothbrushing frequency						
<2	185 (8.1)	149 (12.4)	**<0.001**	279 (8.9)	55 (14.7)	**0.005**
≥2	1904 (91.9)	940 (87.6)		2542 (91.1)	302 (85.3)	
Regular dental visit						
No	1381 (66.2)	751 (66.6	0.819	1885 (66.2)	247 (67.9)	0.627
Yes	708 (33.8)	338 (33.4		936 (33.8)	110 (32.1)	
Hypertension						
No	1540 (80.5)	609 (61.5)	**<0.001**	2008 (77.4)	141 (45.3)	**<0.001**
Yes	549 (19.5)	480 (38.5)		813 (22.6)	216 (54.7)	
Obesity						
No	1451 (71.3)	648 (58.6)	**<0.001**	1908 (68.8)	191 (52.5)	**<0.001**
Yes	638 (28.7)	441 (41.4)		913 (31.2)	166 (47.5)	
Hypercholesterolemia						
No	1850 (88.4)	870 (78.2)	**<0.001**	2461 (86.9)	259 (67.4)	**<0.001**
Yes	239 (11.6)	219 (21.8)		360 (13.1)	98 (32.6)	

Values are presented as number (weighted percent). * *p*-value obtained from chi-square test. † *p*-value obtained from *t*-test. Bold denotes statistical significance at *p* < 0.05.

**Table 2:** Distribution of hs-C Reactive protein according to the periodontitis and diabetes mellitus (N = 3178).

	Periodontitis			Diabetes Mellitus		
Variables	No (*n* = 2089)	Yes (*n* = 1089)	*p*-Value *	No (*n* = 2821)	Yes (*n* = 357)	*p*-Value *
hs-CRP (quartile)						
I (0–0.30 mg/L)	570 (28.3)	208 (19.1)	**<0.001**	727 (26.7)	51 (12.5)	**<0.001**
II (0.31–0.55 mg/L)	478 (23.7)	253 (25.5)		668 (25.0)	63 (16.2)	
III (0.56–1.00 mg/L)	546 (25.4)	298 (26.2)		740 (25.3)	104 (29.2)	
IV (1.10–21 mg/L)	495 (22.7)	330 (29.2)		686 (23.1)	139 (42.1)	
hs-CRP (ADA/CDC)						
Low risk (<1.0 mg/L)	1534 (74.8)	717 (66.9)	**<0.001**	2050 (74.2)	201 (53.4)	**<0.001**
Average risk (1.0–3.0 mg/L)	394 (18.3)	269 (24.5)		565 (19.3)	98 (30.3)	
High risk (3 mg/L)	161 (6.8)	103 (8.6)		206 (6.5)	58 (16.3)	
hs-CRP (mg/L), mean ± SE	1.16 ± 0.06	1.35 ± 0.09	**<0.001 †**	1.13 ± 0.04	2.21 ± 0.28	**<0.001 †**

Values are presented as number (weighted percent). * *p*-value obtained from chi-square test. † *p*-value obtained from *t*-test. Bold denotes statistical significance at *p* < 0.05.

**Table 3:** Adjusted association of hs C-reactive protein levels with periodontitis and diabetes mellitus co-existence (N = 3178).

		Periodontitis–Diabetes Mellitus Co-Existence				
		G1: perio(-)DM(-)	G2: perio(+)DM(-)	G3: perio(-)DM(+)	G4: perio(+)DM(+)	
Variables	n	(*n* = 1911)	(*n* = 910)	(*n* = 178)	(*n* = 179)	*p*-Value
hs-CRP (quartile)						
I (0–0.30 mg/L)	778	29.1 (1.6)	20.8 (1.8)	15.9 (3.1)	9.3 (2.3)	<0.001 *
II (0.31–0.55 mg/L)	731	24.1 (1.3)	27.0 (1.7)	16.2 (3.0)	16.2 (3.0)	
III (0.56–1.00 mg/L)	844	25.0 (1.3)	25.9 (1.5)	30.7 (4.2)	27.7 (3.9)	
IV (1.10–21 mg/L)	825	21.8 (1.2)	26.3 (1.8)	37.3 (4.9)	46.8 (4.0)	
hs-CRP (ADA/CDC)						
Low risk (<1.0 mg/L)	2251	75.8 (1.2)	70.1 (1.8)	59.2 (5.0)	47.6 (4.1)	<0.001 *
Average risk (1.0–3.0 mg/L)	663	17.8 (1.1)	23.0 (1.5)	26.8 (4.2)	33.7 (4.5)	
High risk (>3 mg/L)	264	6.4 (0.6)	6.9 (1.1)	13.9 (3.5)	18.6 (3.4)	
Unadjusted						
hs-CRP (mg/L)	3178	1.11 ± 0.49 a	1.18 ± 0.08 a	2.04 ± 0.43 b	2.37 ± 0.38 bc	**0.001 †**
Adjusted						
hs-CRP (mg/L)	3178	1.37 ± 0.11 ac	1.28 ± 0.15 ac	2.01 ± 0.37 abc	2.31 ± 0.39 bc	**0.020 ‡**

* Obtained from chi-square test. † Unadjusted mean and standard error by ANOVA. ‡ Adjusted mean and standard error are controlled for age, sex, income, education, smoking, drinking, physical activity, toothbrushing frequency, regular dental visit, hypertension, obesity, and hypercholesterolemia by ANCOVA. Bold denotes statistical significance at *p* < 0.05. Letters a–c show which groups differ significantly; same letters mean no difference, different letters mean significant difference.

**Table 4:** Adjusted Odds ratio (95% CI) for the association between periodontitis and diabetes mellitus co-existence and hs-CRP levels (N = 3178).

		Periodontitis and Diabetes Mellitus Co-Existence			
		Model 1	Model 2	Model 3	Model 4
Variables	*n*	G1: perio(-)DM(-)	G2: perio(+)DM(-)	G3: perio(-)DM(+)	G4: perio(+)DM(+)
hs-CRP (quartile)					
I (0–0.30 mg/L)	778	Reference	Reference	Reference	Reference
II (0.31–0.55 mg/L)	731	0.83 (0.62–1.10)	1.28 (0.96–1.71)	0.82 (0.46–1.44)	1.18 (0.59–2.36)
III (0.56–1.00 mg/L)	844	1.02 (0.79–1.31)	0.92 (0.69–1.21)	1.24 (0.72–2.14)	1.74 (0.92–3.28)
IV (1.10–21 mg/L)	825	0.84 (0.64–1.10)	0.91 (0.66–1.24)	1.52 (0.80–2.90)	2.54 (1.387–4.68) *
hs-CRP (ADA/CDC)					
Low risk (<1.0 mg/L)	2251	Reference	Reference	Reference	Reference
Average risk (1.0–3.0 mg/L)	663	0.83 (0.66–1.04)	1.00 (0.79–1.28)	1.33 (0.80–2.19)	1.77 (1.14–2.75) *
High risk (>3 mg/L)	264	0.89 (0.62–1.28)	0.67 (0.44–1.01)	1.78 (0.93–3.41)	2.73 (1.64–4.54) *

CI: confidence interval. All models were adjusted for age, sex, income, education, smoking, drinking, physical activity, toothbrushing frequency, regular dental visit, hypertension, obesity, and hypercholesterolemia. * Bold denotes statistical significance at *p* < 0.05.

## Data Availability

The data used for this study are openly available to the public on request from the KNHANES website (https://knhanes.cdc.go.kr/knhanes/eng/index.do; accessed on 2 December 2024).
